# Patient Reflections on Participation in a Randomised Controlled Multimodal Prehabilitation Trial Before Ventral Hernia Repair

**DOI:** 10.3390/ijerph22071039

**Published:** 2025-06-30

**Authors:** Sofie Anne-Marie Skovbo Jensen, Siv Fonnes, Jacob Rosenberg, Hanne Tønnesen, Susanne Vahr Lauridsen

**Affiliations:** 1 Clinical Health Promotion Centre, WHO-CC, the Parker Institute, Bispebjerg and Frederiksberg Hospital, 2000 Frederiksberg, Denmark; sofie.anne-marie.skovbo.jensen.01@regionh.dk (S.A.-M.S.J.); susanne.vahr.lauridsen.02@regionh.dk (S.V.L.); 2 Centre for Perioperative Optimization, Department of Surgery, Herlev Hospital, University of Copenhagen, 2730 Herlev, Denmark; 3 Department of Clinical Medicine, University of Copenhagen, 2200 Copenhagen, Denmark; 4 Department of Health Sciences, Faculty of Medicine, Lund University, 22100 Lund, Sweden

**Keywords:** qualitative study, prehabilitation, ventral hernia repair, lifestyle, risk reduction, minor surgery

## Abstract

Background: The aim was to explore patients’ reflections related to their choice of participating or not in a multimodal prehabilitation randomised controlled trial (RCT) in relation to minor surgery. Methods: A qualitative study with 22 semi-structured in-depth interviews on patients awaiting ventral hernia repair was conducted between March and May 2024 and reported according to the COREQ guideline. All were eligible to participate in a prehabilitation RCT; twelve had accepted, and ten had declined. The interviews were analysed using Kirsti Malterud’s method of systematic text condensation, resulting in themes. Results: Five global themes were identified: “time commitment”, “research participation for the general good”, “personal benefits of RCT participation”, “ambivalence of own health and lifestyle”, and “complications after surgery”. All participants found the RCT and its prehabilitation programme a positive initiative. Those who had accepted to participate in the RCT emphasised personal benefits and contributing to research, while those who had declined expressed more ambivalence regarding lifestyle change, the extent of personal advantage, and prioritising of time. Conclusions: Those who declined RCT participation generally had more elaborate and ambivalent reflections than those who had accepted. Addressing ambivalence regarding lifestyle change, personal benefits, and prioritising time might be a relevant focus point for increasing inclusion rates in prehabilitation RCTs and in clinical practice to increase patients’ readiness for lifestyle change.

## 1. Introduction

Smoking, Nutrition (both obesity and malnutrition), risky Alcohol intake, and Physical inactivity (SNAP) are known to increase the risk of postoperative complications after both minor and major surgery [1,2,3,4,5,6,7], and prehabilitation interventions targeting these have been evaluated in different studies [8,9,10,11]. However, recruitment to randomised controlled trials (RCTs) testing the effect of lifestyle interventions before surgery has been challenging both internationally [12,13] and in a Danish setting [14], i.e., the setting of this study. RCTs in general often have difficulties with recruitment [15], and getting patients to engage in and actually change their lifestyle can also be challenging [16]. It is therefore not surprising that RCTs of preoperative lifestyle interventions face recruitment challenges, as they require patients to be willing to both participate in research and to actively engage in their health and healthcare. Consequently, gaining insight into what matters to patients in deciding whether to participate or not in a prehabilitation RCT is important.

This understanding can be advanced through qualitative studies, which can provide in-depth insights into patients’ reflections, values, and decision-making processes. Existing qualitative studies have primarily explored barriers and facilitators from patients’ and healthcare professionals’ perspectives, especially towards physical exercise [17,18,19,20,21] and smoking cessation interventions [22]. Close to all interview studies on prehabilitation have been conducted on patients going through life-changing processes, such as having a cancer diagnosis or being frail while facing major surgery [17,18,19,20,21,23,24]. Getting a cancer diagnosis has previously been described as “a window of opportunity” to change lifestyle [25]. In contrast, patients preparing for minor elective surgery, e.g., procedures with expected short duration times, minor resections, and short recovery times, may not perceive the same urgency or motivation. As such, their reflections on prehabilitation and prehabilitation trials may differ significantly from those of patients facing more serious conditions. To our knowledge, no one has explored patient perspectives towards multimodal prehabilitation targeting one to five SNAP factors before elective minor surgery. Moreover, we will be the first to include perspectives from patients who were invited to participate in a prehabilitation RCT but declined. Gaining these insights is important for developing prehabilitation programmes in clinical trials to which patients will accept participation. Furthermore, it may help develop programmes which are meaningful to patients and feasible to implement if proven effective in the trials.

This study aimed to explore patients’ reflections related to their choice of participating or not in a multimodal prehabilitation RCT in relation to minor surgery.

## 2. Materials and Methods

### 2.1. Study Design

This qualitative study, based on semi-structured in-depth interviews, was reported in compliance with the consolidated criteria for reporting qualitative research (COREQ) [26] and approved by the Danish Data Protection Agency (P-2024-15598). No approval from the Danish Ethical Committee was necessary due to the qualitative design [27]. All participants received oral and written information about the study and signed an informed consent form before being interviewed. We obtained the interviews and applied the method of systematic text condensation by Kirsti Malterud [28] to gain knowledge about the reflections of patients with ventral hernia towards a multimodal prehabilitation programme and the choice of participating or not in a clinical trial.

### 2.2. Participants and Setting

Interviews were conducted between March and May 2024 with patients scheduled for elective ventral hernia repair at Herlev Hospital, Denmark, a public university hospital where all healthcare services are free. The inclusion criterion for this interview study was that the participants had to be eligible for the STRONG-Hernia prehabilitation RCT [29]. It was an ongoing RCT aiming to investigate a prehabilitation programme individualised to the specific patient’s needs and targeting all five risky SNAP factors (the STRONG programme) compared with treatment as usual.

The participants were approached by project personnel immediately after they visited the outpatient clinic where they were scheduled for surgery. If eligible for the STRONG-Hernia prehabilitation RCT, they were also informed about the aim of this interview study and invited to participate. Both patients who declined and those who accepted RCT participation were invited. Inclusion criteria for the RCT and this interview study included being scheduled for ventral hernia repair, having at least one risky SNAP factor, and having strong language skills in either Danish or English [29]. The main exclusion criteria included hernia defect width > 8 cm, having surgery scheduled in less than 4 weeks, pregnancy/breastfeeding, and severe mental illness. Participants were provided with the information in Table 1 about the STRONG programme during the RCT recruitment process. All interviews were conducted face-to-face. The participants were offered to be interviewed either in the hospital or online in a video meeting.

### 2.3. Sampling Strategy

We used the principle of purposeful sampling with maximum variation [30], thereby targeting participants who varied in sex, age, and whether they were working or retired. We considered the concept of Information Power [31] when assessing sample size. Our narrow aim, as well as the participants being highly specific for the research question and with variation in their characteristics (dense sample specificity), led to a smaller sample size. Likewise, the ability of the participants to communicate their experiences and opinions reflectively influenced the sample size [32]. Still, conducting a cross-case analysis and not applying an established theory for grounding the conclusions pointed towards a larger sample size needed to achieve sufficient information power. Based on these considerations and previous experiences in the author group with similar qualitative studies, a number of 10–14 participants per group, i.e. 10–14 participants who had accepted RCT participation and a further 10–14 who had declined, was deemed a probable number to achieve sufficient information power. The quality of the interviews in relation to the research questions and whether interviews continued to contribute new relevant knowledge was assessed continuously.

### 2.4. Data Collection

The authors had prepared a semi-structured interview guide with broad, open-ended questions and follow-up questions to use in all interviews (see Supplementary Material File S1). The opening question was “What were your thoughts when you heard about the research project’s programme to change lifestyle?”. Both the interviewer and the senior researcher (SVL) had been interviewed beforehand to clarify their preconceived assumptions and biases regarding the participants and their potential reflections. All but two of the interviews were conducted by the first author (SASJ), who presented herself as a doctor and PhD student. She had no previous experience with conducting interviews and qualitative research. Her preconception interview included the following notions: (1) patients would potentially decline RCT participation due to the high time consumption in relation to hernia surgery being a minor procedure; (2) making lifestyle changes is difficult, especially when one is overweight.

A senior researcher (SVL) with previous experience in conducting qualitative studies listened to the recordings and read the transcripts of the first two interviews to supervise the interview technique. A research colleague with previous interview experience conducted two of the interviews and was present at two of the interviews conducted by the first author. She presented herself as a nurse researcher, supervising the interviews. At two of the interviews, the spouse of the participant was present. The rest was conducted without others present.

Audio recording was used for all interviews. Field notes were made right after the interview. Then, the audio files were transcribed into text with artificial intelligence (Aiko app for Mac, https://apps.apple.com/us/app/aiko/id1672085276) (accessed on 9 April 2024) and subsequently listened to thoroughly with manual corrections to gain an exact transcription of the interview word for word. Transcripts were not returned to participants for comments or corrections afterwards. Initial steps of analysis were conducted after the first two interviews and again after four interviews. The interview guide was adjusted continuously. No repeat interviews were conducted.

### 2.5. Data Analysis

**Figure 1:**
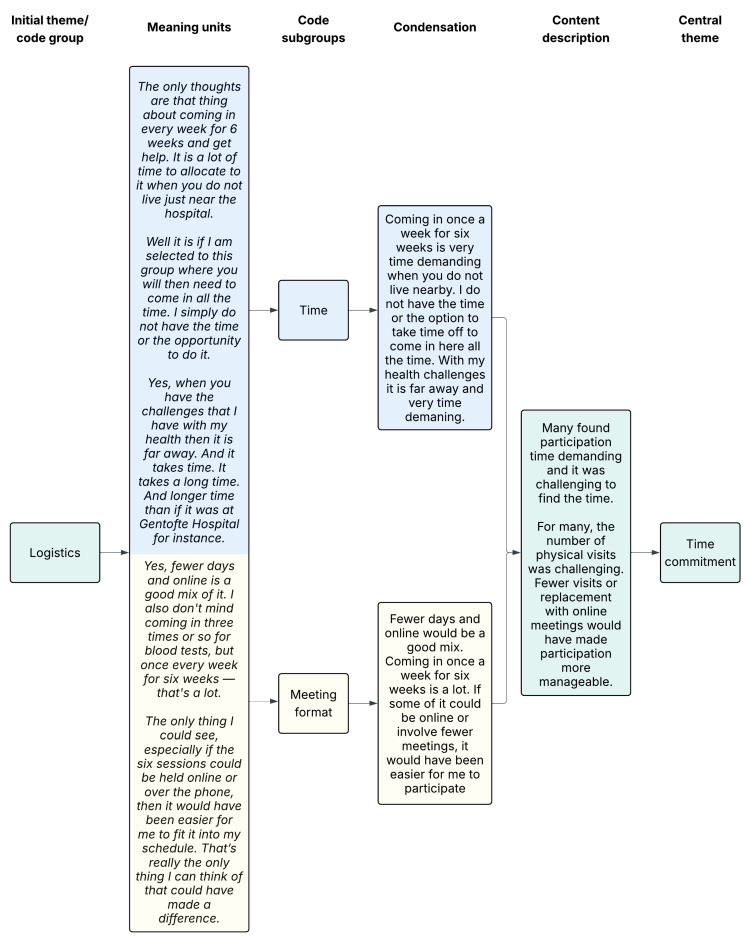
Example of coding tree. The colors illustrate how data progresses from a shared starting point (green), through subcodes that are processed separately (blue and yellow indicate different subcodes, their corresponding meaning units, and condensations), and is then brought back together into a unified description and central theme (green).

We used systematic text condensation by Malterud [28], i.e. a thematic cross-case analysis method, to analyse data and derive themes. The interviews for participants who had declined and accepted RCT participation, respectively, were analysed separately and then compared in the end. First, SASJ and SVL each read the interviews with an open mind to get a total impression of the data. Then they discussed the interviews and decided on preliminary themes to use for the coding, derived from an inductive approach. Secondly, meaning units were identified, classified, and sorted into codes in an iterative process supported by the NVivo 14 software^®^. Thirdly, the data were reduced into condensates within each code group (and subgroups), and relevant quotations were identified. Finally, data were reconceptualised by resynthesizing condensates within a code group into an analytic text. The category heading was also reconceptualised, thereby deciding on the central theme. An example of the coding tree can be seen in Figure 1. Finally, the two analyses were compared. Parts of the first two interviews were coded in collaboration by SASJ and SVL. Afterwards, the remaining was coded by SASJ with support from SVL when needed. The participants did not provide feedback on the findings.

### 2.6. Trustworthiness

To ensure that the study was trustworthy, we considered credibility, transferability, dependability, and confirmability as described by Guba [33] and Shenton [34]. A lot of provisions have been made to add credibility to the study. Examples included the use of a well-recognised research method, iterative questioning, and triangulation by conducting semi-structured interviews and using field notes with observations from the interviewer about the interview. The research group included both experts in the patient group and the intervention group. Debriefing sessions were held continuously between the interviewer and the senior researcher, and an examination of previous research was conducted to frame and discuss the findings.

To enable the readers to assess transferability to other patients with ventral hernia, participant characteristics were reported. A description of the RCT and the intervention was included, so the potential transferability to other prehabilitation programmes or clinical trials on patients undergoing minor surgery could be assessed. To address the issue of dependability, an in-depth description of the methodology was applied to enable the study to be repeated. This also added to confirmability. Finally, we used quotations when presenting the findings.

## 3. Results

We invited 12 participants who had accepted to participate in the prehabilitation RCT (RCT accepters) and 16 who had declined RCT participation (RCT decliners). All 12 RCT accepters consented to interviews, while 10 of the 16 RCT decliners accepted being interviewed. Of the remaining six, three declined due to lack of interest or mental capacity, two initially accepted but were subsequently unresponsive, and one underwent surgery before the interview could be conducted. Data saturation was agreed upon after 10 and 12 interviews, respectively.

Baseline characteristics are reported in Table 2. The participants included fifteen men and seven women aged 34 to 87 years. Half were working and half were retired. The SNAP factors varied among the participants, but more than half were obese. In both groups, some had a history of previous surgical interventions, while others had never undergone surgery before. Eleven participants were interviewed at the hospital right after giving consent, seven came in for the interview another day, and four participants preferred the video meeting as the setting. The interviews varied from 16 to 37 min, with a median duration of 24 min.

The general attitude toward the prehabilitation RCT and the STRONG programme was very positive, and all interviewed found it both interesting and of value. However, the RCT decliners mentioned most considerations, barriers and challenges. The analysis resulted in five themes, all including expressions from both groups. In the following, the RCT accepters were referred to as P-YES while the RCT decliners were referred to as P-NO.

### 3.1. Time Commitment

This was a major theme for the P-NO. They described study participation as time-demanding, which was problematic for different reasons and, in general, a barrier to participation. The P-YES prioritised the participation, expressed willingness to invest the time, and, overall, the time demand did not take up much thought in their reflections regarding the choice of participating.

*“Logistics. The question is whether I can find the time for potentially weekly visits. But as long as there is some flexibility on this end, then I am also willing to make the time.”*

(P2 YES, 43-year-old man)

For the P-NO, those with a loaded everyday life due to children, full-time jobs, long travel time, and/or healthcare problems expressed that it could be challenging to allocate and prioritise the time.

*“You could say that what ultimately made me decline was simply time. I’m a mother of young children, and I have a full-time job with several responsibilities.”*

(P2 NO, 39-year-old woman)

Both groups reflected on whether physical attendance was necessary for all trial visits or if some could be replaced with online or telephone meetings to save time. Many of the P-NO found the number of physical visits challenging, whereas it was not a big issue for the P-YES.

### 3.2. Research Participation for the General Good

A major theme among the P-YES was their general positive attitude towards research and their wish to contribute. Hearing that it was a research project was often enough for them to say yes. Some recognised that someone had to take part because if everyone declined, there would be no development. A sense of civic responsibility or even duty was expressed. Many P-YES also mentioned a desire to do good for others. They hoped the project would lead to valuable findings that could benefit themselves, others, and the healthcare system.

*“Well, it was because someone has to! We can’t all say no to participating… Otherwise, there won’t be any progress. (…) Without sounding too self-righteous, it’s just basic civic responsibility.”*

(P6 YES, 68-year-old man)

The P-NO also expressed gratitude that someone took an interest in conducting research to benefit patients, which they, in general, would like to contribute to. Nevertheless, it did not seem to take up much of their reflections or impact on whether to accept or decline RCT participation.

### 3.3. Personal Benefits of RCT Participation

Another theme for both groups was the personal advantage of participation in the RCT, although the two groups had varying perspectives. When introduced, the impression in both groups was positive, seeing it as a valuable opportunity for lifestyle change. The P-YES identified personal benefits such as potential health improvements, enhancing the chance of a better surgical course, and, for smokers, a financial incentive.

*“My thought was that after speaking with the doctor, it made sense that I was recommended a robotic surgery, and the idea that the less we have here (pointing towards stomach), the better it probably is, right? So, I thought it was a good idea because I could take action myself before the surgery (…).”*

(P8 YES, 74-year-old man)

The P-NO had more elaborate and ambivalent reflections. While many saw potential health benefits, and some mentioned enhancing the chance of a better surgical course as a plus, many also felt the RCT was not relevant for them, or they did not need the support to change their lifestyle. Some were confident to do it alone, others had successfully performed it before or had already begun making changes to their lifestyle and, therefore, saw no need to participate. Common for all was that the personal benefits of participating did not seem significant enough.

*“So, combined with what I think I would gain from it, in relation to the things I am already doing, it was a decision on my part to opt out.”*

(P3 NO, 48-year-old man)

### 3.4. Ambivalence of Own Health and Lifestyle

When asked about a healthy body, some focused on healthy lifestyle factors. However, in both groups, many described a healthy body as one that enables them to carry out daily activities with minimal discomfort while maintaining an overall sense of physical and mental well-being.

*“It is not necessarily a slim body. For me, it is a body that works where the musculoskeletal system does not hurt. And that you can do things without too much discomfort. In other words, having a decent everyday life—do some gardening, exercising, taking a bike ride without having to lie down afterwards and groan…”*

(P5 NO, 60-year-old woman)

Both groups often experienced a sufficiently satisfying everyday life, since they could often still perform most daily activities they valued. The P-NO often emphasised their medical conditions rather than their risky lifestyle as the primary influences on their daily lives. In contrast, the P-YES were more evenly balanced in what impacted their daily life.

*“Well, of course, you can feel that you’re carrying a few extra kilos, but uh… Sometimes. But otherwise I’d say I feel fine. I mean, the only issue with those extra kilos is that you get more out of breath, also because you don’t move around as much as you used to. But otherwise, I’d say I feel fine.”*

(P4 YES, 58-year-old man)

Nevertheless, the P-YES still saw room for improvement and desired to quit their risky behaviours. Some P-NO stated that they had learned to live with their situation, while others proudly shared how they had begun making changes and felt improvements in their health and daily functioning.

*“I know that I’m too heavy, and I know I should lose weight, because I know I can’t keep up with my kids anymore, but I don’t have pain (…) I don’t mind being big. So I actually feel healthy enough, even though I know, according to my BMI and all that, I know I’m not.”*

(P1 NO, 34-year-old woman)

When asked what had previously prevented them from changing their lifestyle, many P-YES found it difficult to change their lifestyle effectively or lacked the motivation. The P-NO had various reasons for continuing their lifestyle. Some mentioned knowing what changes they needed to make, but found it difficult to implement or sustain them. Others felt unable to make changes at the moment due to a lack of either mental or physical capacity. A few simply did not wish to change.

### 3.5. Complications After Surgery

As all participants were scheduled for surgery, they had a discussion with a surgeon about potential complications related to the surgery. Nevertheless, they did not all express clear knowledge on this. The P-YES exhibited varying levels of knowledge about surgical complications and how risky lifestyles increase postoperative risks. The P-NO generally described having limited knowledge about this or having never considered it. However, their reflections were often unclear or ambiguous. Some initially denied any knowledge of the connection, but later recalled hearing that smoking could impair healing or, less commonly, that obesity might complicate surgery. Still, they frequently struggled to understand the link or when it applied.

*“I don’t know anything about that. I did not even know that it affected anything at all. Because I wasn’t told anything like that at all when I had my prostate surgery back then, so… (…) The doctor said that I should… What is it called… Well… That it would be a good idea to cut down on tobacco and alcohol a few weeks before surgery. And also after so you… I mean both… So there would be a better healing process.”*

(P6 NO, 61-year-old man)

Both groups expressed little concern about developing surgical complications and the increased risk associated with their risky lifestyle. Some P-YES felt the hernia repair was necessary despite the risk of potential complications. Many P-NO expressed little to no fear of complications, largely because they had not given it much thought. Instead, some expressed fearing other aspects, such as undergoing general anaesthesia, and some mentioned previous surgeries without complications provided a sense of reassurance. In addition, participants from both groups expressed a generally optimistic and pragmatic outlook as a reason for not worrying about the risk of complications.

*“No, I’m not afraid. I have to take things as they come. If it… Well, life isn’t without risks in general … So, what’s the word… I expect it will turn out fine. If it doesn’t, that sucks. But on the other hand, there’s probably a way forward. There’s no need to expect the worst—you might as well take a positive approach.”*

(P11 YES, 83-year-old man)

Finally, participants in both groups added confidence in the healthcare system, the doctors, and medical science as a reason for not worrying about their upcoming surgery.

## 4. Discussion

Across the groups, five global themes related to their choice of participating or not in a multimodal prehabilitation RCT were identified: time commitment, research participation for the general good, personal benefits of RCT participation, ambivalence of own health and lifestyle, and complications after surgery. While both groups viewed the RCT as a positive opportunity, RCT accepters emphasised personal benefits and contributing to research, whereas RCT decliners expressed greater ambivalence, particularly regarding lifestyle change, extent of personal advantage, and prioritising of time. Neither of the groups was too concerned about developing surgical complications.

It is well-established that RCTs in general may have difficulties with recruitment and reaching the targeted sample size necessary to create more robust evidence [15], and that changing lifestyle is challenging [16]. Therefore, this RCT was double-challenged. Patient concerns about time commitment or reluctance to prioritise participation have also been reported in other qualitative prehabilitation studies in relation to major surgery [19,20,24]. Similar findings have been reported in non-prehabilitation settings as well [35]. The time demand can be difficult to alter, but the perception of it or the importance of prioritising participation for the patients may be something to focus on to increase inclusion rates. Consistent with findings from other non-prehabilitation RCTs [35], contributing to research and helping others were facilitating factors among those who accepted participation in our RCT. However, it held little significance in the reflections of those who declined. The two themes, “personal benefits of RCT participation” and “ambivalence of own health and lifestyle,” could suggest the importance of making patients reflect on and recognise the potential personal benefits of prehabilitation and changing lifestyle. The first theme showed a distinction in perceptions between the two groups. RCT accepters saw clear personal benefits, whereas RCT decliners expressed more ambivalence about the personal relevance and benefit. This aligns with findings from other qualitative studies [19,21,22,23,24]. The second theme showed surprisingly many common perceptions in the two groups. They shared similar views on a healthy body and daily life satisfaction, and ambivalence about their health. However, RCT accepters more clearly recognised a need and desire for lifestyle change than the RCT decliners. A study on prehabilitation reported low inclusion rates before ventral hernia repair, with time as one of the barriers and patient ambivalence about health, similar to our findings [36]. However, participation rates rose markedly after integrating a survey on patients’ health and desire for risk modification into the enrollment process [36]. Together with our findings, this suggests prehabilitation participation rates could possibly increase by encouraging self-reflection or educating patients on how their risky lifestyles raise surgical complication risks. The final theme showed a general low concern about complications across both groups. When being scheduled for surgery, all had received information from a surgeon in the outpatient clinic on the risks of surgery, but despite this, many expressed not knowing a lot. Especially, the RCT decliners were characterised by not having reflected much on the risk of complications or association with risky lifestyles. This indicates that educating patients and prompting reflection on the impact of risky lifestyle factors on surgical risk, even in minor elective procedures, may be relevant to support recruitment.

We did not assess the readiness to change lifestyle in the participants [37], but interestingly, the results of the analysis also fit well into the framework of the stages of change model. It describes the six stages one is expected to go through when changing a behaviour [38]. They include precontemplation, contemplation, preparation, action, maintenance, and termination. Progressing through the stages should not be seen as a linear process, but as a cyclic process. The time needed to progress through stages and how many times to go through the cycle can differ between individuals. The RCT accepters seemed more determined and with fewer reflections than the RCT decliners. This fits into the preparation or action stage, where the person is ready to make the change. The ambivalence present in many of the RCT decliners regarding the personal benefits and relevance of participation, as well as the importance of lifestyle change, suggests they may be in the contemplation stage. This stage is characterised by changing their attitudes and weighing the pros and cons before taking the next step towards changing behaviour. Ambivalence should not be seen as a lack of motivation, but rather as a sign of considering the change; in other words, they have begun the process of change [38]. Therefore, participants expressing great ambivalence could be the most important to engage in dialogue with and educate on health, risks of surgery, and the link with their risky lifestyle to spark self-reflections, decrease the cons, and increase the pros of making a lifestyle change. Some will stay in this stage for a longer period, while others get ready to progress to the preparation or action stage [38]. Furthermore, the framework also provides guidance for professionals to understand how to support the patients’ progress through the stages. It indicates that support for people in the initial stages is education and information; this could be more important to focus on when trying to recruit patients with ambivalence rather than support and encouragement [38].

The strengths of this qualitative study included several authors being involved in creating and revising the interview guide as well as the initial coding. Also, both the interviewer and the supervising senior researcher were interviewed before conducting any interviews to clarify their preconceptions. This aided them in being as neutral as possible when interviewing the participants and analysing the interviews. Despite her inexperience, the PhD student obtained in-depth answers by using prompts from the interview guide and repeating questions to stay focused on the research question. The quality of dialogue was deemed strong when the senior researcher listened to initial interviews and read the transcripts as part of the initial review of data and supervision of the PhD student. Finally, the study was reported according to COREQ to ensure high quality and transparency. A classic limitation of qualitative studies, including this study, is the limited transferability. Qualitative interview studies usually obtain in-depth insights into a somewhat context-specific phenomenon through small sample sizes. This interview study was conducted on a selected and narrow population of patients awaiting elective ventral hernia repair and investigated reflections towards a specific intervention, being prehabilitation of risky lifestyle factors. Therefore, a limitation is that transferability only applies to a similar population faced with a similar intervention in a similar setting. In this study, the results can be transferred to other patients with ventral hernia facing similar prehabilitation interventions. However, it could be argued that the results may also be transferred to populations undergoing other minor surgical procedures than ventral hernia repair. Finally, transferability should be considered carefully as the study furthermore took place in a high-income country with a free public healthcare system. Noting whether patients were scheduled for open or laparoscopic procedures could also have enhanced the interpretability and aided in assessing transferability of the findings to other settings. As a second limitation, the reflections of the RCT decliners may not fully represent the broader population of patients who decline participation in a prehabilitation RCT, as those who declined to be interviewed may hold different views. Thirdly, identification of “time commitment” as a global theme may have been impacted by the interviewer’s preconception that the time demand could be a barrier to participation, which questions the credibility. However, both groups were interviewed by the same person, and the RCT accepters did not perceive the time demand as an obstacle. Instead, they expressed a willingness to prioritise the time, reinforcing the credibility of this theme. Fourthly, information on participants’ previous surgical history was not collected and was not included in the semi-structured interview guide. As a result, the potential influence of prior surgery on their perceptions of the upcoming hernia repair and lifestyle change was not systematically explored in all patients. In retrospect, this would have been a valuable addition to the interview guide, as previous surgical experience may shape patients’ attitudes toward both surgery and preoperative lifestyle modification. Finally, a limitation was that the participants did not get the opportunity to comment on the transcripts.

This study highlighted that allocating the necessary time was a significant barrier to participating in the prehabilitation RCT. While the time demand itself may be difficult to change significantly, our findings also included other more modifiable factors impacting the choice of participation which may offer potential actionable targets for intervention such as the patients’ perception of the importance of prioritising time for prehabilitation, the benefits of changing lifestyle before surgery, and awareness of the risks of surgery and association with unhealthy lifestyles. Although the findings are context-specific and not universally transferable, they are highly relevant to similar surgical settings. From a health promotion perspective, these insights may inform strategies to improve patient recruitment in future similar prehabilitation RCTs and encourage reflection on clinical practice in similar surgical settings. Specifically, the ambivalence present in many of the RCT decliners, in contrast to the RCT accepters, might suggest that engaging in dialogue with ambivalent patients to promote self-reflection and interest may enhance their readiness to change and adopt healthier behaviours preoperatively. This might not only contribute to improving individual outcomes but also to supporting broader public health goals by increasing prehabilitation research participation, reducing lifestyle-related surgical risks, and potentially lowering complication rates and healthcare costs.

## 5. Conclusions

This qualitative study found that patients who had declined participation in the prehabilitation RCT expressed more ambivalence toward making lifestyle changes at the moment, the perceived personal benefits of participation, and prioritising the time than those who accepted. RCT accepters were, in contrast, more motivated by potential health benefits and contributing to research. Knowledge about surgical risk and its connection to lifestyle varied, but few participants in either group were concerned about complications. Specifically, RCT decliners had not given it much thought. Providing targeted education and information to ambivalent patients during recruitment might be part of a relevant strategy to help increase inclusion in prehabilitation RCTs. While the findings are context-specific, and transferability therefore should be considered carefully, they may inform RCT recruitment strategies and encourage reflections on clinical practice to increase patient readiness for lifestyle change in similar or related surgical settings.

## Figures and Tables

**Table 1:** The information that eligible participants received about the STRONG Programme.

All participants	
Six meetings before surgery would be held at the hospital, i.e., on a weekly basis Participants receive education and motivational and pharmaceutical support (optional, free) Blood and urine samples are collected at some of the meetings	
**Specific interventions according to the risky lifestyle**	
Smokers:	Personalised nicotine replacement therapy in accordance with participant preferences and nicotine dependency
Risky drinkers:	Optional: ○ Thiamine and B vitamins ○ Alcohol withdrawal prophylaxis and treatment ○ Disulfiram
Obese or at risk of malnutrition:	Personalised nutritional plan, Nupo™ meal replacements (optional)
Physically inactive:	Personalised physical exercise plan

**Table 2:** Baseline characteristics of participants.

Characteristics		RCT Decliners (*n* = 10)	RCT Accepters (*n* = 12)
Sex, *n* (%)			
	Men	7 (70)	8 (67)
	Women	3 (30)	4 (33)
Age, median [range]		61 [34–84]	69 [40–87]
Working, *n* (%)			
	Yes	5 (50)	6 (50)
	No	5 (50)	6 (50)
Risky lifestyles, *n* (%)			
	Daily smoker	3 (30)	4 (33)
	BMI > 30 kg/m^2^	6 (60)	6 (50)
	Malnutrition (NRS ≥ 3)	0 (0)	1 (8)
	Alcohol intake > 14 units/week	1 (10)	3 (25)
	Physically active < 30 min/day	2 (20)	3 (25)
Number of risky lifestyles, *n* (%)			
	1 risky lifestyle	8 (80)	8 (67)
	2 risky lifestyles	2 (20)	3 (25)
	≥3 risky lifestyles	0 (0)	1 (8)

BMI: body mass index, *n*: number, NRS: nutritional risk score; RCT: randomised controlled trial.

## Data Availability

The datasets presented in this article are not readily available due to the presence of personally identifiable information in the interview transcripts. Sharing them would compromise participant confidentiality and could violate data protection regulations.

## Supplementary Materials

The following supporting information can be downloaded at: https://www.mdpi.com/article/10.3390/ijerph22071039/s1, Supplementary File S1: Translated interview guide.

## Abbreviations

COREQ	Consolidated criteria for reporting qualitative research
P-NO	Participants who declined to participate in the prehabilitation RCT
P-YES	Participants who agreed to participate in the prehabilitation RCT
RCT	Randomised controlled trial
SNAP	Smoking, nutrition (both obesity and malnutrition), alcohol, and physical inactivity
