# Supplementary material for: Patient Reflections on Participation in a Randomised Controlled Multimodal Prehabilitation Trial Before Ventral Hernia Repair
Source: Int J Environ Res Public Health. 2025 Jun 30;22(7):1039. doi: 10.3390/ijerph22071039 (PMC12294419; doi:10.3390/ijerph22071039)
Supplement: Supplementary file 1 [file ijerph-22-01039-s001.zip › ijerph-3658716-supplementary.pdf]

# Supplementary Materials

## Supplementary file S1. Translated interview guide

<table>
<tr><td colspan="3">Aim/objectives<br><br>To explore patients’ reflections related to their choice regarding participation in a multimodal prehabilitation trial in relation to minor surgery</td></tr>
<tr><td colspan="3">Indledning<br><br>We appreciate that you will help us gain more knowledge about the thoughts of patients undergoing ventral hernia repair on their own health, our programme on changing lifestyle, and choice regarding participation in a research project. It means a lot.<br><br>The interview has a structure with some open-ended questions and some follow-up questions, but there is room for us to talk about the subjects that are important to you.<br><br>First, we will ask some general baseline questions, and then we will ask the actual questions about health, the programme, and the research project.</td></tr>
<tr><td>Subject</td><td>Question</td><td>Prompts and follow-up questions</td></tr>
<tr><td>Reflections towards the STRONG programme</td><td>What were your initial thoughts when you heard about the research project’s programme to changing lifestyle?</td><td>• What advantages of the research project’s programme to changing lifestyle do you see?<br>• What disadvantages of the programme do you see?<br>• Ideally, how should the programme be structured for you?<br>• Do you have any suggestions to changes in the programme?<br>• What do you think the programme can help with that you are/have been unable to do yourself?</td></tr>
<tr><td>Reflections regarding the choice of participating or not in the STRONG-Hernia trial</td><td>What were your reflections regarding deciding whether to participate in the STRONG-Hernia trial or not?</td><td>• What advantages did you see of participating in the research project?<br>• What disadvantages or challenges did you see of participating?</td></tr>
</table>

| | | |
|---|---|---|
| | | • What determined your choice to participate or not?<br>• Can you elaborate?<br><br>If ”no” to participation:<br>• What would it have taken for you to say yes to participate? |
| View on own health and the choice of participation or not | What is a healthy body to you? | • How would you describe your own health?<br>• What do you think about your lifestyle?<br>• How do you see that your current lifestyle affects your health?<br>• What has prohibited you from changing your lifestyle?<br>• How do your reflections on your health and lifestyle affect your choice regarding participation in the trial? |
| Link between lifestyle and complications | What do you know about lifestyle and the risk of getting complications in relation to your surgery? | • Can you elaborate?<br>• What information did you receive from the doctor?<br>• What did you know in advance?<br>• Are you scared of getting complications after your ventral hernia repair?<br>o If yes: Why? Can you elaborate?<br>o If no: Can you elaborate? |
| End of interview | Do you have anything else to add before we end the interview? | |

**Ending**

Thank you for today. I am really thankful that you participated in this interview and appreciate your contribution to research. It means a lot.
